# PSgANet: Polar Sequence-Guided Attention Network for Edge-Related Defect Classification in Contact Lenses

**DOI:** 10.3390/s26020601

**Published:** 2026-01-15

**Authors:** Sung-Hoon Kim, In Joo, Kwan-Hee Yoo

**Affiliations:** Department of Computer Science, Chungbuk National University, Cheongju 28644, Republic of Korea; sidsid84@gmail.com (S.-H.K.); injoo95@naver.com (I.J.)

**Keywords:** artificial intelligence, contact lens, manufacturing quality control, polar coordinate transformation, sequence learning, attention guided network

## Abstract

The integration of artificial intelligence (AI) into industrial processes is a promising method for enhancing operational efficiency and quality control. In particular, contact lens manufacturing requires specialized artificial intelligence technologies owing to stringent safety requirements. This study introduces a novel approach that employs polar coordinate transformation and a customized deep learning model, the Polar Sequence-guided Attention Network (PSgANet), to improve the accuracy of defect detection in the rim-connected zone (RCZ) of contact lenses. PSgANet is specifically designed to process polar coordinate-transformed image data by integrating sequence learning and attention mechanisms to maximise the capability for detecting and classifying defective patterns. This model converts irregularities along the edges of contact lenses into linear arrays via polar coordinate transformation, enabling a clearer and more consistent identification of defective regions. To achieve this, we applied sequence learning architectures such as GRU, LSTM, and Transformer within PSgANet and compared their performances with those of conventional models, including GoogleNetv4, EfficientNet, and Vision Transformer. The experimental results demonstrated that the PSgANet models outperformed the existing CNN-based models. In particular, the LSTM-based PSgANet achieved the highest accuracy and balanced precision and recall metrics, showing up to a 7.75% improvement in accuracy compared with the traditional GoogleNetv4 model. These results suggest that the proposed method is an effective tool for detecting and classifying defects within the RCZ during contact lens manufacturing processes.

## 1. Introduction

Artificial intelligence (AI), one of the core technologies of the Fourth Industrial Revolution, has become a means of reducing organisational costs and enhancing service quality, coordination, and productivity [1]. Furthermore, AI has been regarded as the most innovative technology over the past three decades and is expected to bring about extensive changes across various industries. With advancements in machine learning, which allow computers to perform specific functions by learning from data, AI research has been widely applied across multiple fields [2].

In manufacturing industries, integrating AI into quality inspections can significantly enhance process efficiency. AI vision inspection, which combines conventional machine vision technology with artificial intelligence, enables more accurate and efficient product inspection. This technology can automatically distinguish between acceptable and defective products using image data acquired during the inspection process. Compared with manual inspection processes, AI vision inspection can perform these tasks faster and more efficiently [3]. When AI technologies are applied to manufacturing processes, various improvements can be anticipated that directly influence productivity and defect rates. Therefore, AI technologies for manufacturing must be highly reliable, precise, and capable of real-time operations, distinguishing them from conventional AI technologies [4]. Moreover, industries requiring high precision and safety increasingly depend on specialised AI technologies for quality management. Contact lens manufacturing is a prime example of this. Contact lenses, medical devices worn on the cornea, have revolutionised ophthalmology and vision correction [5,6]. Owing to convenience and continuous advances in materials and design, the use of contact lenses is steadily increasing [7,8,9]. Ensuring the safety and effectiveness of these lenses is crucial not only for optimal vision correction but also for preventing potential ocular complications [10,11,12].

Quality control standards for contact lenses are specified in ISO 18369-1:2017 [13], and the fundamental requirements for contact lenses are detailed in ISO 14534:2015 [14,15]. These standards not only cover overall quality management requirements but also deeply explore specific standards and responsibilities related to various areas of contact lenses. The manufacturing process of contact lenses involves multiple stages utilising hydrophilic polymer materials. These stages include injection molding as part of the forming process, tinting, reagent filling, and assembly, followed by separation, drying inspection, hydration inspection, sealing, sterilisation, and packaging. Minor anomalies or inconsistencies occurring at any stage are detected and classified during the hydration inspection process [16,17,18,19,20,21,22,23]. In the manufacturing process, the hydration inspection step is crucial for quality assurance, particularly for detecting and removing defects. Detecting defects in areas that directly contact the eye is critical [24,25,26].

Automated optical inspection (AOI) systems have been introduced to detect and classify defects in contact lenses. These systems comprise illumination, cameras, and image processing algorithms designed specifically for defect detection [27,28]. Studies have shown that these methods yield more accurate results than traditional visual inspection methods. However, the accuracy of conventional image-processing-based algorithms is limited owing to the wide variety of patterns and defects present in contact lenses. Consequently, visual inspection methods utilising deep learning have been introduced to achieve impressive results in image recognition and processing [29]. Nevertheless, existing deep learning-based approaches require further improvement in terms of accuracy and reliability. In particular, for products such as contact lenses, which require high precision, the accurate detection of subtle defects and variations poses significant challenges.

**Figure 1:**
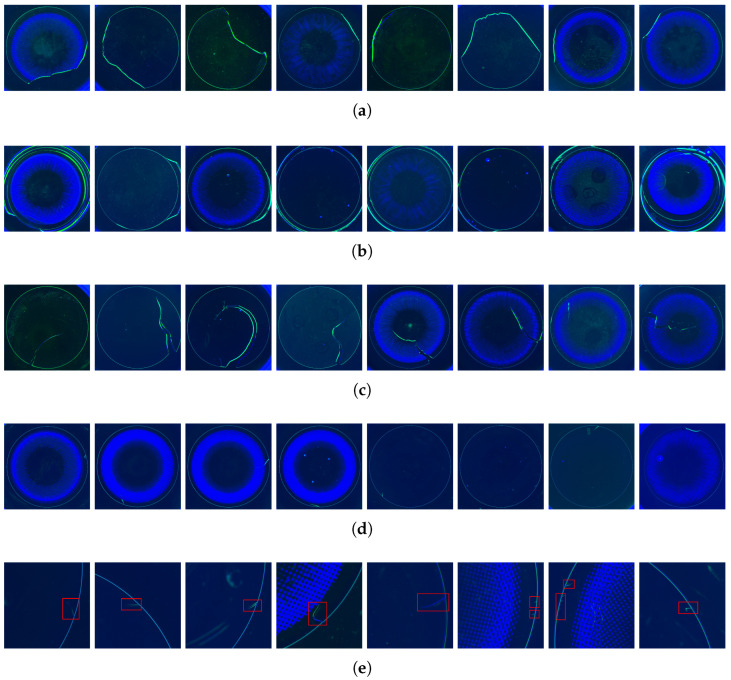
Examples of various edge defect types: (**a**) broken: broken defective. (**b**) burr: edge cutting failure. (**c**) b_edge: large scale edge defect. (**d**) n_edge: normal scale edge defect. (**e**) i_edge: tiny scale edge defect (red frame).

Employing AI-based vision inspection during the hydration inspection stage, which is the final inspection step in the contact lens manufacturing process, is essential to ensure quality management. Figure 1 illustrates the various types of defects observed at the edges of the contact lenses in the images acquired during the hydration inspection process. This is caused by physical damage or material defects that occur during the manufacturing process. In particular, it is critical because it carries the risk that the size of the defect may increase depending on the flow of the manufacturing or distribution processes. They are classified in the order of Figure 1c b_edge > Figure 1d n_edge > Figure 1e i_edge according to size.

- (a) broken: This defect image shows that the lens was so damaged that it was completely detached.
- (b) burr: This example shows a defect that occurs during lens separation, resulting in an unpolished edge. The inner contact lens retains its original shape, but an unnecessary part remains.
- (c) b_edge: Among the edge defects, those with lengths greater than 40% of the lens diameter were classified as b_edge.
- (d) n_edge: This is a typical example of edge defects.
- (e) i_edge: These defects are extremely small, typically 2–5% of the contact lens diameter, and may develop into forms such as n_edge or b_edge during sterilisation, packaging, or distribution processes. Therefore, detecting these defects during the hydration inspection stage is crucial.

In the hydration inspection of contact lenses, it is crucial to detect and classify even the smallest defects. In particular, fine defects such as i_edge, which account for approximately 2–5% of the lens diameter, correspond to only approximately 40–150 pixels when converted to pixel units, making their characteristic detection highly challenging. However, such microdefects are highly likely to develop into more severe forms, such as n_edges or b_ edges, during stabilisation, packaging, or distribution. Therefore, they must be carefully selected and managed during the hydration inspection stage. When the image size is reduced for image processing or deep-learning model training, such microdefects may disappear entirely or suffer from pixel information loss, resulting in undetected defects. Moreover, when detecting small defects using deep learning models, insufficient resolution makes it difficult to extract meaningful features from small defect regions, potentially causing confusion due to background noise. This directly leads to a degraded performance, including false positives, false negatives, or overfitting. Therefore, it is essential to utilise ultra-high-resolution images and design models capable of capturing fine-grained features.

## 2. Related Works and Research Purpose

This section outlines prior research related to defects that occur during the contact lens manufacturing process. We also discuss the limitations of these studies in identifying and addressing edge-related defects in contact lenses.

Zin et al. (2018) proposed a method for classifying the presence of contact lenses by extracting features from the lens edge [30]. In their study, the region of interest was first segmented based on the iris area, and features from the lens edge were extracted using histograms of oriented gradients (HOG) [31] and scale-invariant feature transform (SIFT) [32] algorithms. The extracted features were then classified using a support vector machine (SVM) [33], which identified the decision boundaries between the data groups to determine whether a soft lens was being worn. However, the scope of their study differs from ours, while their research focused on identifying the lens-wearing status to enhance the security of iris recognition systems, our work aims to precisely identify defective products during the manufacturing process of contact lenses, an objective with a fundamentally different direction.

Chunhachatrachai et al. (2023) [34] proposed a novel computer vision algorithm, CLensRimVision, for automatic identification of subtle edge imperfections in contact lenses. The algorithm involves image preprocessing, circle detection, polar coordinate transformation, definition and localisation of the defect criterion, and visualisation of the final result. In particular, it incorporates two approaches, thickness- and area-based, to handle a wide range of edge-related issues with precision. The experimental results demonstrated the effectiveness of the algorithm, achieving an average precision (AP) of up to 0.937. This study is similar to our research goals and employs polar coordinate transformation, making it notably similar. However, their work focused solely on burr-type imperfections within the RCZ, among many other possible defect types, and the evaluation was conducted using only 60 images, which limited data diversity. Therefore, further validation is required to assess the robustness of the visual algorithm.

In the study by Kim et al. [35], convolutional neural network (CNN)-based deep learning models such as ResNet101 [36], GoogLeNetv2 [37], GoogleNetv4 [38], DenseNet121 [39], and MobileNet [40] were used to classify defective contact lenses during the manufacturing process. This study also compared and analysed the classification performance across the RGB and HSV channels. The accuracy scores of each model were reported as 89.74%, 84.46%, 95.43%, 82.80%, and 89.74%, respectively, with GoogleNetv4 exhibiting the highest accuracy in the RGB channel. Most models demonstrated superior performance with RGB over HSV. However, this study focused on identifying and classifying defects related to printing irregularities rather than physical defects in the contact lens itself and therefore differs in scope from our work.

Kim et al. [20] proposed a defect-adaptive hierarchical structure called DHS-CNN and demonstrated accuracy improvements of 1.39% and 2.08% over conventional deep learning models such as GoogleNetv4, EfficientNet [41], and Vision Transformer (ViT) [42] by employing a customised loss function. This study focused on identifying the physical defects during the manufacturing process using an inspection procedure aligned with ours. However, among the defect types illustrated in Figure 1a broken, Figure 1b burr, Figure 1c b_edge, Figure 1d n_edge, and Figure 1e i_edge, this study only addressed broken, burr, and b_edge defects, excluding n_edge and i_edge. As previously mentioned, these finer defects are highly likely to develop into critical issues during the subsequent sterilisation, packaging, or distribution stages; therefore, they must be thoroughly identified and managed during the hydration inspection stage.

Recent advances in deep learning have demonstrated the effectiveness of attention mechanisms in image classification tasks. Hybrid approaches such as FCIHMRT (Feature Cross-layer Interaction Hybrid Method based on Res2Net and Transformer) [43] have shown significant improvements by enabling feature cross-layer interaction and dynamically focusing on relevant features. However, these methods operate on 2D spatial features. Our approach differs by applying attention mechanisms to sequence features extracted from polar-transformed images, which is specifically designed for circular defect patterns in contact lens inspection.

This issue warrants particular attention, given its potential severity. Minor defects can escalate into critical problems during manufacturing or distribution, making their prompt identification and management essential. The rim area of a contact lens is extremely sensitive, and imperfections in this region not only cause discomfort during wear but also lead to tear film disruption or even damage to the ocular surface [34]. Therefore, it is crucial to develop a new AI-based approach capable of more precise inspection to overcome the limitations of existing models.

**Figure 2:**
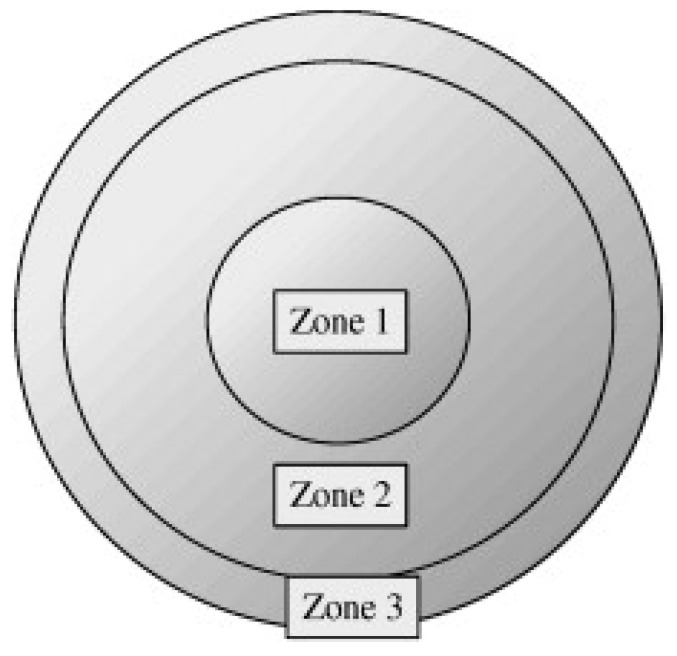
Contact lens area classification [15].

Figure 2 shows the structural zones of the contact lenses. The innermost circular area is referred to as the central optical zone, zone 2 as the rim optical zone, and zone 3 as the rim-connected zone (RCZ). Our study focused specifically on the defects occurring within the RCZ, which is considered a safety-critical area for users [34].

The objective of our study was to analyse the image data obtained from the hydration inspection process in contact lens manufacturing with the aim of identifying and categorising various types of defects within the RCZ. Accordingly, our study falls under the classification task of deep learning-based image analysis techniques. Convolutional neural network (CNN)-based models have traditionally dominated the classification tasks; however, vision transformer (ViT) models demonstrated excellent performance in this domain [44].

However, although these methods generally achieve a reasonable level of performance, they are not well suited for direct applications in scenarios such as manufacturing processes that demand high accuracy. Contact lenses have a circular shape with a consistent size and geometry, and edge-related defects typically form along the rim-connected zone (RCZ), which includes the circular boundary of the lens. Therefore, our study aims to improve the classification accuracy of edge defect images by proposing a method that specifically focuses on the circular edge patterns observed in contact lens image data.

It should be noted that while Figure 1 illustrates various defect types to demonstrate inspection complexity, our study formulates the problem as binary classification. The critical question for quality control is not “what type of defect exists?” but rather “does any defect exist in the RCZ?” Therefore, all edge defects are treated as a single “defective” class to maximize detection sensitivity.

The main contributions of this study are as follows:

- Domain-specific problem reformulation: We reformulate circular RCZ edge defect detection as a 1D sequential pattern recognition problem through polar coordinate transformation.
- PSgANet architecture for sequential edge analysis: We design a network combining 1D-CNN, sequence learning modules (LSTM/GRU/Transformer), and weighted attention to process polar-transformed data.
- Improved defect detection performance: Our method achieves 93.66% accuracy with the LSTM-based PSgANet, demonstrating a 7.75% improvement over the baseline GoogleNetv4 model under pixel-count-comparable conditions.

## 3. Proposed Method

**Figure 3:**
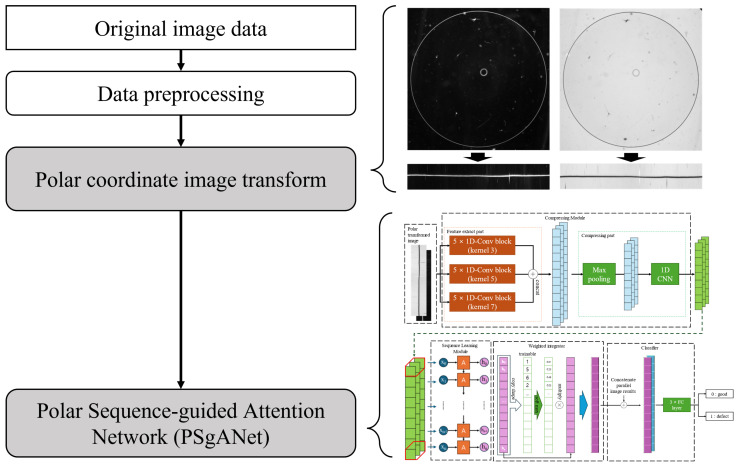
Overall flow of proposed method.

The methodology proposed in this study aims to enhance classification performance by extracting features specifically from the RCZ region of the original image. To achieve this, the process consists of three key stages: image preprocessing, polar coordinate transformation, and the proposed Polar Sequence-guided Attention Network (PSgANet). Figure 3 illustrates the architecture of the proposed approach.

- The original image data, which served as the starting point of the study, consisted of high-resolution greyscale images captured under two different lighting conditions to form paired samples.
- Data preprocessing: The initial processing steps included adjusting the resolution and size of the image and removing unnecessary background.
- Polar coordinate image transformation: This process converts an original image into a polar coordinate system, revealing its unique visual characteristics. This transformation is particularly useful for analysing objects with circular or curved shapes, and plays a crucial role in enhancing the detection accuracy of defective patterns.
- PSgANet: Using the transformed image as the input, the network analyses the data through various deep learning modules and finally classifies whether it is defective. This network learns the patterns of sequence data and emphasises prominent features to maximise classification accuracy.

### 3.1. Original Image Data

The first stage of the proposed method utilises the original contact lens image data captured by a camera in an actual manufacturing environment. The dataset was collected from a contact lens production facility as part of their quality control process. Each sample comprised a pair of images captured under different lighting conditions. Both images were single-channel grayscale images with a resolution of 2048 × 2448 pixels.

**Figure 4:**
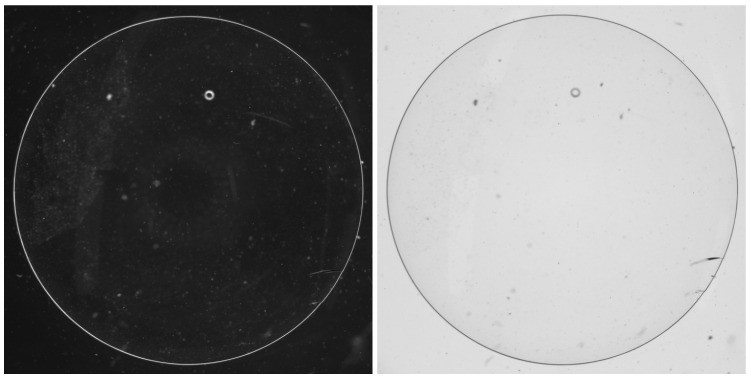
Example of an original pair of images.

As shown in Figure 4, the lighting consists of black and white illumination, designed to acquire defect-related information from multiple perspectives. Even when defects exhibit similar morphological characteristics, variations in lighting or focus can significantly affect visual representation. Therefore, capturing images using the same lens under two contrasting lighting conditions allows for a broader extraction of information that can be leveraged during inspection. Each image pair is acquired from the same lens by switching only the illumination condition between black and white, ensuring spatial correspondence between the paired images.

### 3.2. Data Preprocessing

**Figure 5:**
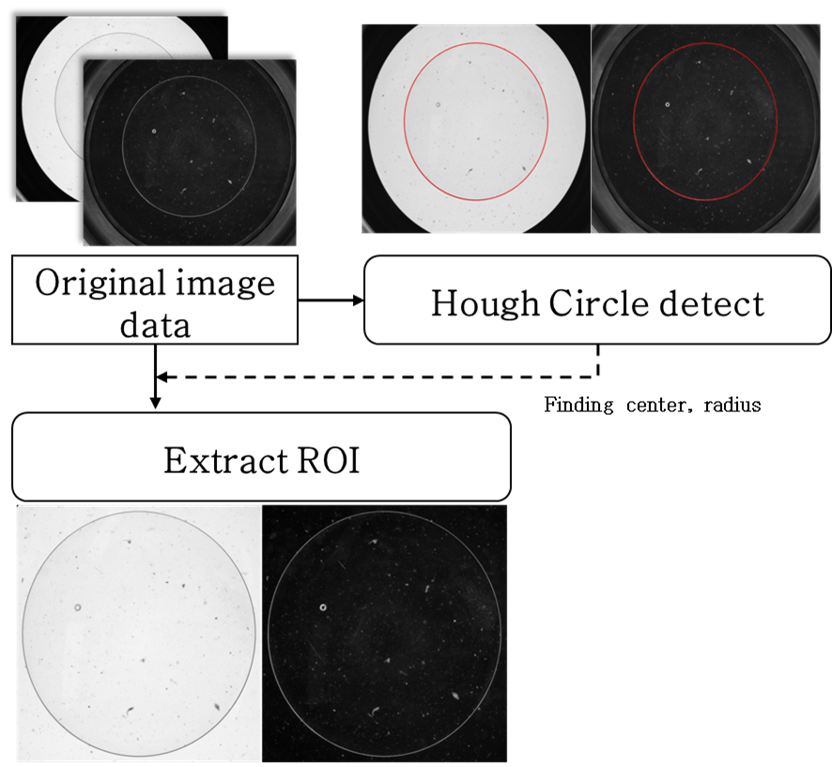
Preprocessing of image data.

The original image described in Section 3.1 has a resolution of 2048 × 2448 pixels. In this study, the accurate extraction of the lens region was crucial for the effective analysis of lens defects. To this end, the Hough Circle Detection algorithm [45] is applied to locate the circular boundary of the lens from which the centre coordinates and radii were precisely determined. The detected radius ranged from 650 to 750 pixels, and based on this information, a 1500 × 1500-pixel square region of interest (ROI) centred on the lens was extracted. This step effectively removed unnecessary background elements from outside the lens area. The entire preprocessing procedure is illustrated in Figure 5.

The square region of interest (ROI) defined relative to the lens centre ensures consistency in the analysis area and contributes to improved accuracy in identifying defect patterns. This consistent focus area allows the model to learn the key characteristics of the data more effectively and distinguish defect types with greater clarity. Moreover, the preprocessing step, which removes the peripheral and background regions, significantly reduces the amount of noise present in the data. This helps minimise the errors caused by irrelevant information and improves the signal-to-noise ratio, thereby enhancing the overall performance of the model. In addition, by eliminating unnecessary regions, the volume of data to be processed is reduced, leading to an increased processing speed and overall computational efficiency. This process enhances the precision of the analysis and improves the operational effectiveness of the system.

The preprocessed ROI data were subsequently used as inputs for the polar coordinate transformation and PSgANet model.

### 3.3. Polar Coordinate Image Transformation

Polar-coordinate transformation is a process that converts an original image into a polar coordinate space and restructures the spatial arrangement of the image to enable the extraction of features from a different perspective. This transformation enhances the visibility of the characteristics near circular boundaries and is particularly effective for objects with circular structures, such as contact lenses.

**Figure 6:**
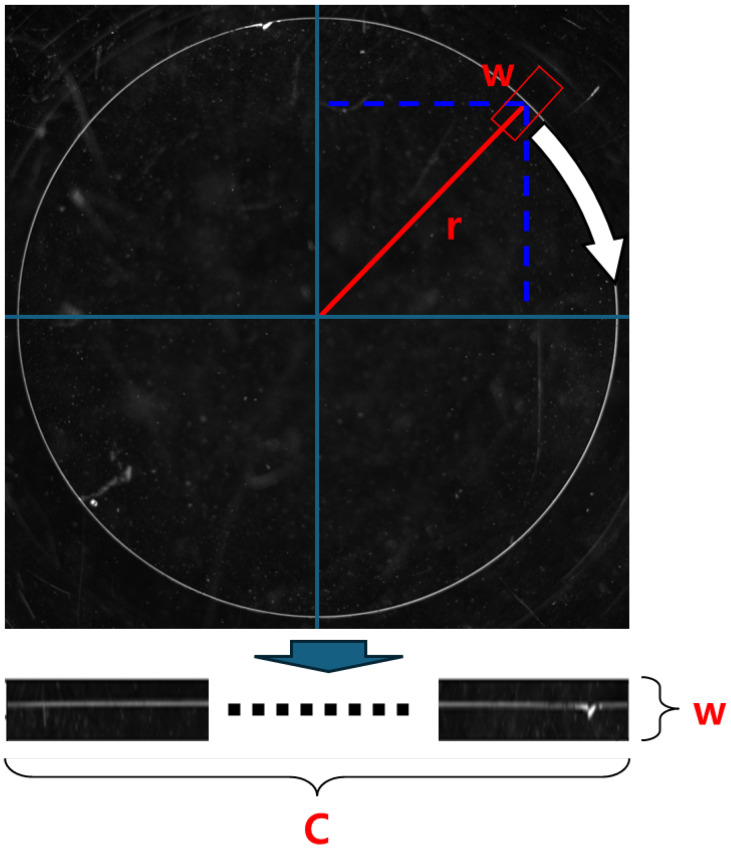
Polar coordinate transformation.

Figure 6 illustrates the transformation process. Here, *r* represents the pixel-wise distance from the image centre to the lens edge, and *w* denotes the width of the RCZ.

Accordingly, the total resolution is defined as 2πwr as shown in Equation (1).

(1) Totalresolution=C×w=2πwr

This value is derived by multiplying the circumference of the circle (angular dimension) by the image height (radial pixel count), and quantitatively represents the resolution of the transformed image in polar coordinates. However, in practical implementations, this theoretical resolution is not directly computed or applied; instead, a grid-sampling technique is utilised. In this approach, a normalised coordinate system was created along the radial and angular directions based on the resolution of the input image. A grid was formed from this coordinate system, which was used to sample the input image and construct the corresponding polar-transformed image [46]. The resolution of the resulting polar image may not perfectly match the mathematically derived value of 2πwr, but the primary objective is to preserve visually important information during the transformation by appropriately adjusting the resolution and mapping.

Accordingly, Algorithm 1 does not implement the mathematical definition literally; rather, it is an optimised mapping method designed to balance the visual fidelity and computational efficiency. Moreover, the algorithm is designed for graphics processing unit (GPU)-based execution, in which functions such as mesh grid, cos, and sin are applied independently to each pixel or data point. In Algorithm 1 , the radius is set to the distance from the center to the ROI corner rather than the detected lens radius to ensure complete coverage of edge defects such as burr, which may extend beyond the nominal lens boundary. This parallelisation on a GPU significantly improves the performance and effectively eliminates bottlenecks observed with CPU-based processing. The grid sampling operation employs bilinear interpolation with border padding mode, which ensures smooth sampling at the boundaries and minimizes aliasing artifacts during the coordinate transformation. While Equation (1). provides a theoretical resolution of 2πr, we use θ=1000 to avoid pixel redundancy that would occur with direct mathematical mapping, achieving optimal information preservation with computational efficiency.
**Algorithm 1** Polar Transformation and Divide Image Algorithm**Require:** input_img (image tensor), center=(x,y), w (RCZ height)**Ensure:** transformed_img  1:**function** PolarTransformAndDivide(input_img, center, w)  2:     $radius\leftarrow \mathrm{dist}\left(\mathrm{center},\;\mathrm{corner}(\mathrm{input}\_\mathrm{img})\right)$  3:     width←width(input_img),height←height(input_img)  4:     θ←linspace(0,2π,width),r←linspace(0,radius,height)  5:     (θ,r)←meshgrid(θ,r)  6:     $x_{t}\leftarrow r\cdot \cos\left(\theta \right)+\mathrm{center}.x,\;y_{t}\leftarrow r\cdot \sin\left(\theta \right)+\mathrm{center}.y$  7:     $x_{\mathrm{norm}}\leftarrow 2\cdot x_{t}/width-1,\;y_{\mathrm{norm}}\leftarrow 2\cdot y_{t}/height-1$  8:     $grid\leftarrow \mathrm{stack}(x_{\mathrm{norm}},\;y_{\mathrm{norm}},\;-1)$  9:     polar_img←grid_sampling(input_img,grid)10:     transformed_img←polar_img[0:w,:]11:     **return** transformed_img12:**end function**

The angular origin θ=0 is defined as the positive x-axis direction in the image coordinate system, following the standard polar coordinate transformation shown in Algorithm 1: $x_{t}=r\cdot \cos\left(\theta \right)+center.x$ and $y_{t}=r\cdot \sin\left(\theta \right)+center.y$. And the angle θ increases in the counter-clockwise direction. Since both black and white illumination images are captured from the same lens with shared center coordinates determined by Hough Circle Detection (Section 3.2), their polar transformations maintain consistent angular alignment. Therefore, no explicit rotational normalization is required, as the sequence learning modules inherently provide rotation invariance by learning defect patterns along the θ axis regardless of absolute angular position.

**Figure 7:**
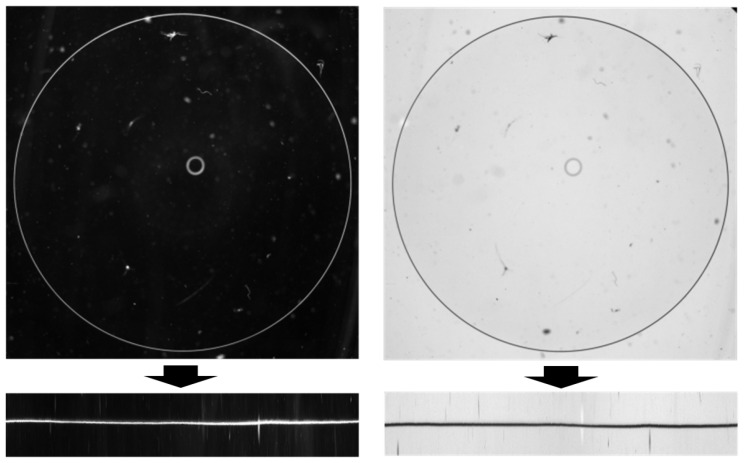
Example of polar transformed image pair.

Through this process, a pair of polar-transformed black and white images was generated, as shown in Figure 7.

### 3.4. Polar Sequence-Guided Attention Network (PSgANet)

**Figure 8:**
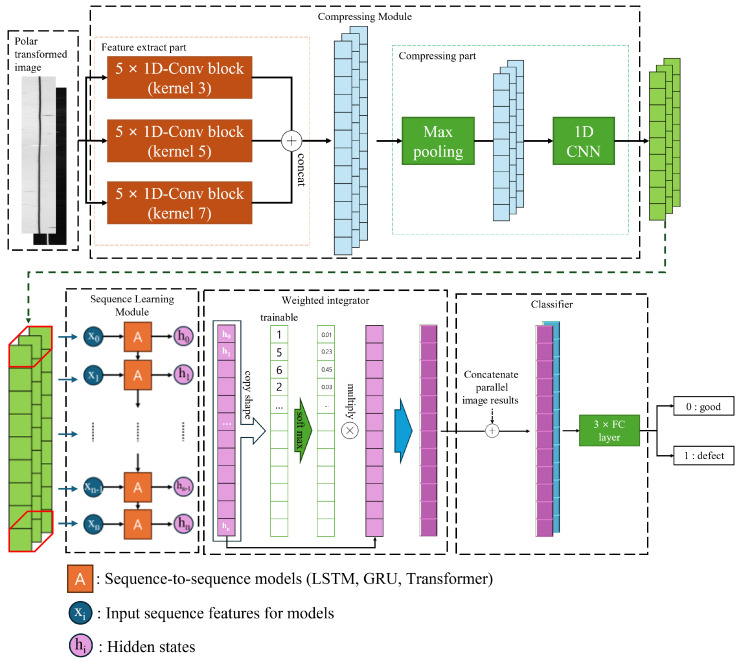
PSgANet architecture.

PSgANet is a deep-learning model specifically designed to effectively analyse and process image data transformed into polar coordinates. As shown in Figure 8, the model comprises four main stages: a compressing module, sequence learning module, weighted integrator, and classifier. This architecture was optimised to facilitate more accurate feature learning from polar transformed images.

- Compressing module: This module extracts essential features from the input data while gradually reducing the number of channels. The objective of this process is to reduce the data complexity and retain only the most relevant information during training.
- Sequence learning module: This module leverages various architectures, such as Long Short-Term Memory (LSTM) [47], Gated Recurrent Unit (GRU) [48], and transformer [49], to learn the sequence structure of data, each of which is specialised in effectively recognising and learning patterns in data with strong temporal or sequential dependencies.
- Weighted integrator: This module performs weighted aggregation to emphasise the most meaningful features extracted by the learning module. It assigns greater weight to key features identified during the sequence learning process, thereby highlighting those that have a significant influence on the overall learning outcome.
- Concat and classifier: Concatenation combines the features generated at various processing stages into a single representative feature vector, which is subsequently used by the final classifier to perform a classification task.

#### 3.4.1. Compressing Module

**Figure 9:**
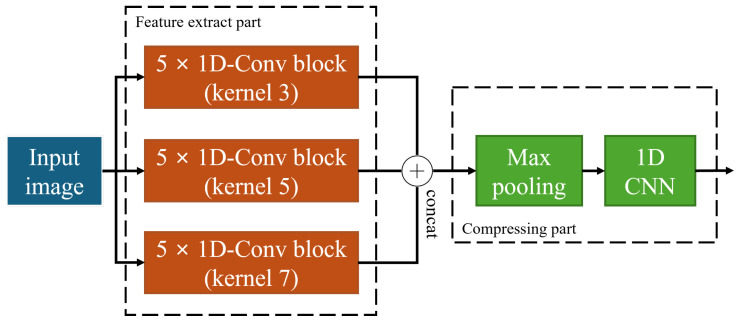
Compressing module architecture.

As shown in Figure 9, the compressing module is divided into two parts, each serving a distinct function. The repeatedly used one-dimensional convolutional neural network (1D-CNN) [50] performs operations along the vertical axis of the image. In other words, it was applied independently to each column of the image, and plays a role in extracting, enhancing, and compressing the features of each column.

- Feature extraction part: The objective of this step was to extract meaningful features while maintaining the original size of the input data. By applying the same padding, the image dimensions were preserved and 1D CNN blocks with kernel sizes of three, five, and seven were each repeated five times. Through these operations, the vertical features of the images were extracted. This approach enables the model to analyse multiple aspects of the data and integrate the extracted features from each stage, deriving comprehensive information [51].
- Compressing part: This process primarily aims to filter irrelevant information and emphasise important features, thereby reducing the dimensionality of the data. In this stage, one-dimensional max pooling with a kernel size of two and a 1 × 1 CNN were applied to significantly compress the feature maps. The resulting number of channels becomes the number of feature dimensions used as inputs to the sequence-learning module.

The compressed data, retaining only the most salient features while eliminating redundant information, were passed to the subsequent sequence-learning module. This not only improves processing speed but also enhances learning efficiency.

#### 3.4.2. Sequence Learning Module

The sequence-learning module was designed to learn the sequential characteristics of the data. This module plays a critical role in identifying and modelling temporal or sequential patterns. Commonly used architectures include LSTM, GRU, and transformers. Each architecture is used to extract essential information from the input sequence and predict or classify the subsequent state based on this information.

In our study, the sequence learning module is particularly important because the compressed edge information forms a sequential structure along the θ direction. The data along the θ axis represents a sequence of visual features, which allows the model to identify repetitive patterns or anomalies within the sequence. This information plays a critical role in how the model processes the data at each point and passes it as an input to the next step. At this stage, the sequence learning module handles the continuous flow of sequential data and updates the information at each point to analyse and understand the overall sequence pattern and characteristics. Although architectures such as LSTM, GRU, and transformers are typically used for future-state prediction, our study focused on analysing local features at each position to identify the presence of specific patterns.

The processed data serve as a critical input for the subsequent stages, enabling the attention mechanism and classification layer to perform more accurate analysis and decision-making based on this information.

#### 3.4.3. Weighted Integrator

**Figure 10:**
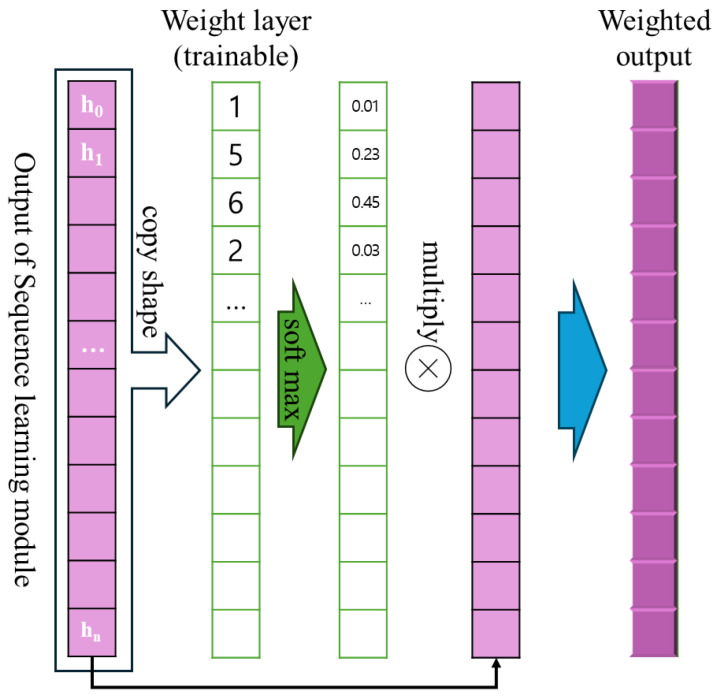
Weighted integrator module operation.

The weighted integrator module comprises an attention mechanism that dynamically evaluates the importance of each sequence element and emphasises the most critical information. The processing flow of this module is illustrated in Figure 10.

First, a learnable linear layer with shared parameters computes attention scores from the input sequence features (i.e., the output described in Section 3.4.2). While the linear transformation parameters are shared across all samples, the attention weights themselves are sample-specific, as they are computed from each sample’s unique sequence features. These weights are then normalized using the softmax function to represent the relative importance of each feature. The weighted output is generated by performing element-wise multiplication between the output of the sequence learning module and the weight layer. This process emphasises the stronger features, resulting in an output in which the most important characteristics are highlighted. This mechanism helps the model to effectively extract and focus on the most relevant information within the entire dataset.

#### 3.4.4. Concat and Classifier

The feature maps extracted from the previous stages—Section 3.4.1, Section 3.4.2 and Section 3.4.3—are processed in parallel for the paired images generated in Section 3.3. Instead of a simple addition operation, concatenation is employed to integrate features while preserving the unique information extracted from each path. These combined feature maps are then passed to a fully connected layer for final classification. In this study, this corresponds to a binary classification task that distinguishes between good and defective samples.

## 4. Experiments

### 4.1. Experiment Environment

The experimental environment used in our study is summarized in Table 1. This configuration was selected to support the training and evaluation of deep learning models on high-resolution contact lens images with polar coordinate transformation.

### 4.2. Dataset

The dataset used in our experiments consisted of pairs of images captured under black- and white-light illumination conditions, with a total of 707 samples: 354 non-defective and 353 defective. The dataset was split at the lens unit level. Lens pairs were divided into training, validation, and test sets, comprising approximately 70%, 10%, and 20% of the data, respectively.

**Figure 11:**
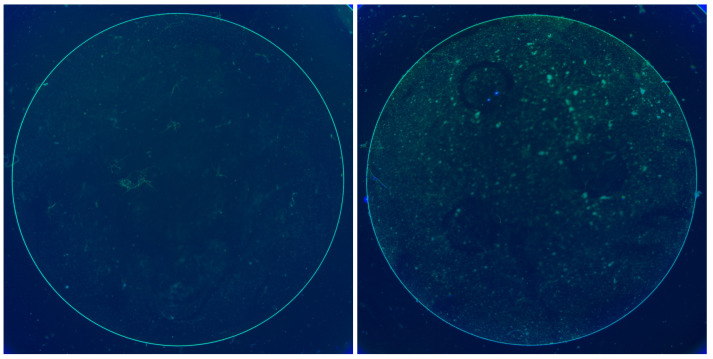
Example of dataset.

Figure 11 shows the sample image pairs from the dataset. We put special effort into making the dataset challenging and deliberately included several difficult cases. For instance, some non-defective lenses have dust or contaminant spots and noise in the images, which can be mistaken for defects, whereas some defective lenses have very subtle edge defects that are difficult to observe. These challenging cases ensured that the model was thoroughly evaluated for its ability to distinguish between true defects and false signals. In the sample images, various artefacts can be observed, and the model must learn to ignore artefacts that are not true edge defects. Each original image had a resolution of 2048 × 2448 pixels, and after preprocessing, a 1500 × 1500 pixel region of interest (ROI) was extracted. The extracted ROI images were transformed into polar coordinates using the method described in Algorithm 1.

Table 2 provides detailed information about the dataset.

### 4.3. Experiment Scenario

In this study, we designed experimental scenarios using four different configurations to evaluate the effectiveness of the sequence-learning module. Each configuration involved training the polar-transformed data using a specific architecture: GRU, LSTM, or Transformer. By applying these different sequence-learning techniques, we conducted a comparative analysis of how effectively each architecture captured and modelled the sequential characteristics of the data. In addition, their performance differences were evaluated by comparing them with traditional CNN-based approaches and transformer-based models, including GoogleNetv4, EfficientNet, and ViT, all of which were applied without polar coordinate transformation. For the baseline models, experiments were conducted at resolutions of 320 × 320, 640 × 640, and 1000 × 1000 pixels, whereas the PSgANet-based models were evaluated at a resolution of 1000 × 106 pixels.

In this context, critical information in the polar coordinate system was determined by the angle (θ) and radius (*r*), where setting θ=1000 indicates that 1000 samples are allocated along the angular direction. This allows for sufficient representation of feature information along the θ axis, while 106 samples are allocated along the relatively less critical radial (*r*) direction. These values were chosen based on the minimum resolution required to ensure reliable performance, as well as considerations of computational efficiency and practical applicability. In addition, to ensure a fair comparison in terms of feature information capacity, the baseline models were evaluated using multiple resolutions: 320 × 320, 640 × 640, and 1000 × 1000.

As shown in Table 3, all the experiments used the same optimisation algorithm (Adam), except for the transformer model, for which the learning rate was set as 0.0001. Binary cross-entropy loss was employed for all models, as the dataset exhibits natural class balance.

To isolate the contribution of polar coordinate transformation from the proposed PSgANet architecture, we evaluated the ViT model with polar transformation at 1000 × 1000 resolution. The polar-transformed images (1000 × 106) used for PSgANet were resized to 1000 × 1000 to create square inputs compatible with ViT. While PSgANet inherently requires rectangular input due to its sequence processing architecture, ViT can accept square inputs, making it suitable for this comparison.

Regarding input resolution fairness, we acknowledge that PSgANet uses 1000 × 106 (106,000 pixels) while baselines use varying resolutions. From a pixel count perspective, 320 × 320 (102,400 pixels) provides the most comparable information content. However, to avoid underestimating baseline performance, we evaluated baselines at higher resolutions (up to 1000 × 1000 = 1,000,000 pixels), deliberately providing them with significantly more pixel information than PSgANet. This conservative approach strengthens our claim that PSgANet’s advantages stem from its architecture rather than information advantage.

This adjustment was made to ensure more stable training because the transformer architecture is known to be highly sensitive to parameter settings. Each experiment was run for more than 100 epochs and early stopping was employed to prevent overfitting. This setup allowed for a systematic analysis of the training process and performance of each model, identification of the optimal sequence learning configuration, and evaluation of the proposed model in comparison with existing approaches.

### 4.4. Experiment Results

For each model, the epoch with the lowest loss during training was selected as the best epoch, and the corresponding weights were used to evaluate the performance of the test set. The evaluation was conducted using precision, recall, accuracy, and F1-score, as defined in Equations (2)–(5).

(2) $$\mathrm{Precision}=\frac{TP}{TP+FP}$$

(3) $$\mathrm{Recall}=\frac{TP}{TP+FN}$$

(4) $$\mathrm{Accuracy}=\frac{TP+TN}{TP+TN+FP+FN}$$

(5) $$F1\text{-}\mathrm{score}\;=2\times \frac{\mathrm{Precision}\times \mathrm{Recall}}{\mathrm{Precision}+\mathrm{Recall}}$$

where TP (True Positive) represents defective samples correctly identified as defective, TN (True Negative) represents non-defective samples correctly identified as non-defective, FP (False Positive) represents non-defective samples incorrectly identified as defective, and FN (False Negative) represents defective samples incorrectly identified as non-defective. In the context of contact lens manufacturing quality control, these metrics carry specific practical significance. Precision measures the proportion of samples identified as defective that are truly defective, directly impacting production efficiency by minimizing unnecessary rejections of acceptable products. Recall measures the proportion of truly defective samples that are successfully detected, which is critical for safety as it ensures defective products do not reach end users. Accuracy provides an overall measure of correct classifications across both classes, while F1-score offers a balanced evaluation by harmonizing precision and recall, making it especially valuable when both false positives and false negatives carry significant costs.

Table 4 presents a comparison of classification accuracy on the test set across various models. The GoogleNetv4 model achieved an accuracy of 85.91% at a resolution of 320 × 320, 78.17% at 640 × 640, and 82.39% at 1000 × 1000. The EfficientNet-B8 model achieved an accuracy of 57.75% at a 640 × 640 resolution. The ViT model achieved 92.96% accuracy at a 640 × 640 resolution and 90.14% accuracy at a 1024 × 1024 resolution. By contrast, the PSgANet model demonstrated superior performance by incorporating the GRU, LSTM, and Transformer architectures. The GRU-based PSgANet model achieved 90.14% accuracy, which is approximately 5% higher than that of the baseline GoogleNetv4 model. The LSTM-based variant achieved the highest accuracy of 93.66%, whereas the Transformer-based PSgANet achieved an accuracy of 91.55%.

**Figure 12:**
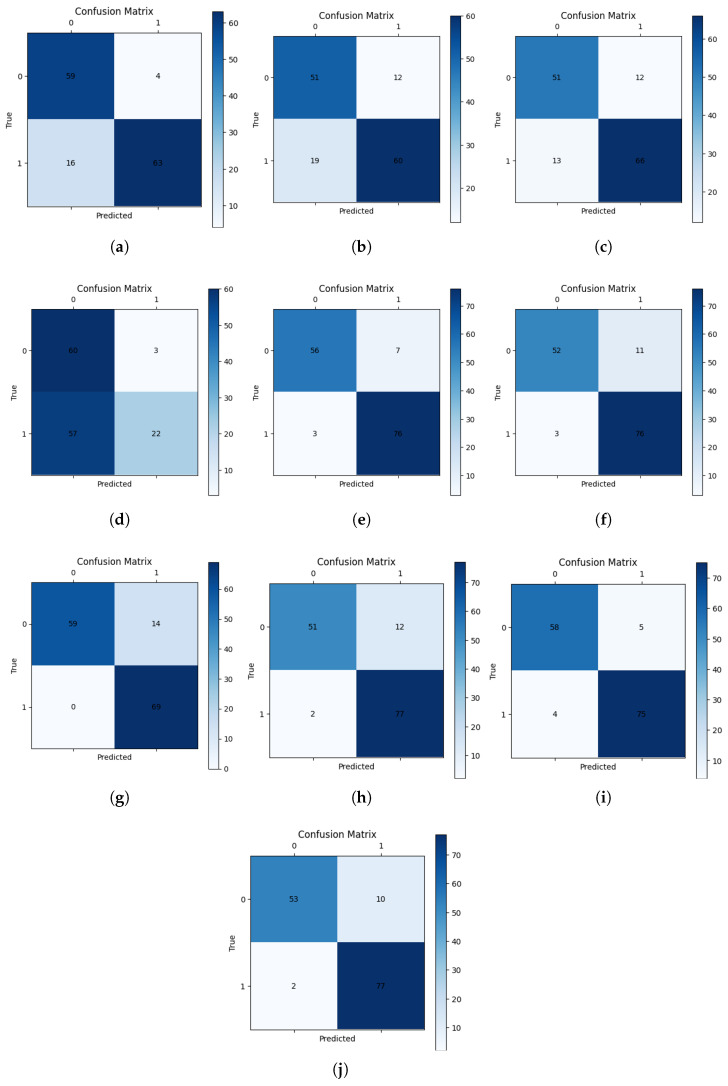
Confusion matrices on the test set (0: good, 1: defect). Panels: (**a**) GoogleNetv4 (320 × 320); (**b**) GoogleNetv4 (640 × 640); (**c**) GoogleNetv4 (1000 × 1000); (**d**) EfficientNet-b8 (640 × 640); (**e**) ViT (640 × 640); (**f**) ViT (1024 × 1024); (**g**) ViT with polar transformation (1000 × 1000); (**h**) PSgANet with GRU; (**i**) PSgANet with LSTM; (**j**) PSgANet with Transformer.

Figure 12 presents the confusion matrices for each model on the test set, where class 0 represents non-defective (good) samples and class 1 represents defective (defect) samples. For the evaluation metrics, class 1 was treated as the positive class, and class 0 as the negative class. GoogleNetv4 with the non-transform model achieved the highest precision; however, its low recall indicates that a sizable number of defective samples were incorrectly identified as non-defective. In contrast, all the other models that used polar coordinate transformation exhibited high recall values. Among them, PSgANet with the LSTM model demonstrated impressive performance in both precision and recall, indicating a well-balanced and reliable classification capability.

**Figure 13:**
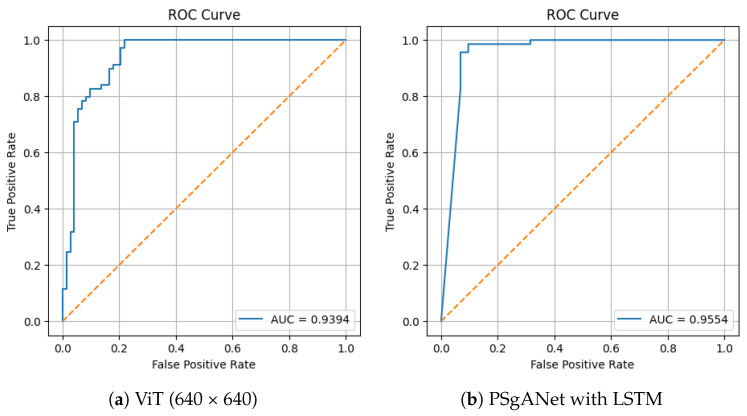
ROC curve for ViT (640 × 640) and PSgANet with LSTM. The orange dashed line denotes the baseline (random guess), indicating an AUC of 0.5.

To further evaluate model performance, we computed the Receiver Operating Characteristic (ROC) curve for the best-performing model, PSgANet with LSTM and ViT (640 × 640). As shown in Figure 13, the model achieved an Area Under the Curve (AUC) of 0.9554, indicating excellent discriminative capability between defective and non-defective samples. This AUC value indicates that the model has a 95.54% probability of correctly distinguishing between a randomly chosen defective and non-defective sample, demonstrating excellent discriminative capability.

## 5. Conclusions

In this study, we proposed a polar-coordinate transformation-based image preprocessing approach and a corresponding optimised model, PSgANet, and evaluated its performance. The experimental results showed that all variants of PSgANet outperformed conventional CNN-based models, particularly those with higher recall values. Recall is critical in manufacturing quality control because a low recall means that defective products can be missed. This metric is critical for quality control strategies in the manufacturing industry.

Among the tested models, PSgANet with LSTM achieved the highest accuracy of 93.66%. To ensure fair evaluation, we compared PSgANet (1000 × 106, 106,000 pixels) against baselines at multiple resolutions. The 7.75% improvement over GoogleNetv4 (320 × 320, 102,400 pixels) represents the combined benefit of polar transformation and specialized architecture under pixel-count-comparable conditions. We also evaluated ViT with polar transformation, where the polar-transformed images (1000 × 106) were resized to 1000 × 1000 (1,000,000 pixels), achieving 90.14% accuracy. This confirms that the performance gain stems primarily from PSgANet’s sequence-aware architecture rather than preprocessing alone. PSgANet-LSTM also demonstrated a well-balanced trade-off between precision and recall.

This suggests that LSTM’s strong capabilities in sequence learning and managing long-term dependencies significantly contribute to its performance. Additionally, the Transformer-based PSgANet exhibited high sensitivity to hyperparameters, implying that its performance could potentially be further improved through optimisation techniques such as grid search. These results confirm that the proposed approach is highly effective for identifying and classifying defects in the RCZ region of contact lenses.

While our study demonstrates the effectiveness of the proposed approach, we acknowledge several limitations and directions for future work. First, although the current results are promising, formal statistical significance testing (e.g., paired *t*-tests across multiple random splits) would further strengthen the validity of our findings, which we plan to include in future research. Second, systematic ablation experiments on polar transformation resolution parameters (θ and *w*) would provide deeper insights into the optimal configuration for different defect scales. Third, while our study formulates the problem as binary classification to maximize detection sensitivity, future work could incorporate stratified analysis by defect size categories (i_edge, n_edge, b_edge) to provide more detailed insights into the model’s sensitivity across different defect scales. This would require collecting additional data with fine-grained annotations for each defect category.

Furthermore, for practical industrial deployment, several implementation considerations warrant attention. Model lightweight design techniques such as knowledge distillation, pruning, or quantization could be explored to reduce computational requirements while maintaining detection accuracy, making the system more suitable for real-time in-line inspection with cost-effective hardware. Additionally, evaluating the model across various image resolutions will be essential for assessing its performance on data of varied sizes and optimizing the model accordingly.

Finally, although our model demonstrated excellent performance in identifying and classifying contact lens defects within the prepared dataset, evaluating its effectiveness in real-world manufacturing scenarios remains a critical challenge. To address this issue, we plan to collect a larger volume of data from diverse production environments and use it to assess and improve the generalisation capability of the model. This comprehensive approach will ensure that the proposed method transitions successfully from research validation to practical industrial application.

## Figures and Tables

**Table 1:** Specifications of hardware and software used in experiments.

Type	Specification
Hardware	CPU: Intel Xeon(R) Silver 4216
	RAM: 240 GB
	GPU: GeForce RTX 3090 24 GB × 2
Software	OS: Ubuntu 20.04
	Language: Python 3.10.4
	CUDA: 11.3
	PyTorch: 1.11

**Table 2:** Dataset details for the experiment.

Description	Details
Total images	707 (defect: 353, non-defect: 354)
Dataset split	Train: 495 (70%), validation: 70 (10%), test: 142 (20%)
Original resolution	2048 × 2448 pixels
Preprocessed resolution	1500 × 1500 pixels (ROI)
Input size for model	1000 × 106 pixels

**Table 3:** Experiment scenarios with various sequence learning modules.

Model Configuration	Resolution	Total Pixels	Learning Rate	Epochs
GoogLeNet-v4	320 × 320	102,400	0.001	100+
GoogLeNet-v4	640 × 640	409,600	0.001	100+
GoogLeNet-v4	1000 × 1000	1,000,000	0.001	100+
EfficientNet-b8	640 × 640	409,600	0.001	100+
ViT	640 × 640	409,600	0.001	100+
ViT	1024 × 1024	1,048,576	0.001	100+
ViT with polar transformation	1000 × 1000	1,000,000	0.001	100+
PSgANet with GRU	1000 × 106	106,000	0.001	100+
PSgANet with LSTM	1000 × 106	106,000	0.001	100+
PSgANet with Transformer	1000 × 106	106,000	0.0001	100+

**Table 4:** Classification accuracy, precision, and recall for the test set.

Method	Precision	Recall	Accuracy	F1-Score
GoogleNetv4 (320 × 320)	**94.03%**	79.75%	85.91%	0.863
GoogleNetv4 (640 × 640)	83.33%	75.95%	78.17%	0.794
GoogleNetv4 (1000 × 1000)	84.62%	83.54%	82.39%	0.841
EfficientNet-b8 (640 × 640)	88.00%	27.85%	57.75%	0.423
ViT (640 × 640)	91.57%	96.20%	92.96%	0.938
ViT (1024 × 1024)	87.36%	96.20%	90.14%	0.916
ViT with polar				
transformation (1000 × 1000)	83.13%	**100.0%**	90.14%	0.908
PSgANet with GRU	86.52%	97.47%	90.14%	0.917
PSgANet with LSTM	93.75%	94.94%	**93.66%**	**0.943**
PSgANet with Transformer	88.51%	97.47%	91.55%	0.928

**Bold** values indicate the best performance for each evaluation metric.

## Data Availability

The data used in this study were provided under a corporate agreement and are subject to confidentiality obligations. Due to company policy and contractual restrictions, the dataset cannot be shared publicly or upon request. The authors do not have permission to distribute the data.

## Abbreviations

The following abbreviations are used in this manuscript:

AI	Artificial Intelligence
RCZ	Rim-Connected Zone
CNN	Convolutional Neural Network
LSTM	Long Short-Term Memory
GRU	Gated Recurrent Unit
ViT	Vision Transformer
PSgANet	Polar Sequence-guided Attention Network
ROI	Region of Interest
AOI	Automated Optical Inspection
